# Expression of HIV from a 1-LTR circular DNA in the absence of integration

**DOI:** 10.1186/s12977-025-00658-1

**Published:** 2025-03-17

**Authors:** Corrado Gurgo, Claudio Fenizia, Katherine McKinnon, Ru-ching Hsia, Genoveffa Franchini

**Affiliations:** 1 Animal Models and Retroviral Vaccines Section, National Cancer Institute, National Institutes of Health, Bethesda, MD 20892 USA; 2 Department of Pathophysiology and Transplantation, University of Milan, Milan, Italy; 3 Electron Microscopy Laboratory, Frederick National Laboratory for Cancer Research, NCI, NIH, Bethesda, MD USA

**Keywords:** Linear 1-LTR, Circular 1-LTR, 1-LTR, HIV

## Abstract

**Background:**

Like all retroviruses, two kinds of viral DNA are present in the nucleus of HIV-infected cells: integrated DNA and a pool of unintegrated DNA containing linear and circular forms. For the most part, it has been difficult to examine the role of the unintegrated DNA forms in the viral life cycle in the presence of the integrated form, or to distinguish the respective contributions of the two circular DNA forms in the context of the unintegrated DNA.

**Results:**

In our approach, we constructed a 1-LTR circular form of HIV in order to study its expression in isolation from the other forms; we derived a linear genomic HIV DNA lacking the 5’-LTR (1-LTR_HIV_) from a molecular clone of HIV. This linear form is transcriptionally incompetent, but via circularization becomes a transcriptionally competent 1-LTR circle. When transfected into cells lacking CD4 where neither the spread of virus nor reinfection can occur, the linear or in vitro circularized form produces a fully infectious HIV. Virus expression is stable throughout cell division as measured on a per cell basis by flow cytometry. A progressive accumulation of copies of the circular form is observed in the presence of the cell growth inhibitor aphidicolin, suggestive of episomal amplification, for which we propose a model.

**Conclusion:**

We demonstrate in this study that production of infectious virus is initiated and completed by the 1-LTR episomal form of HIV DNA in the absence of reinfection and integration. In addition, we show that the 1-LTR episomal form replicates in the absence of an origin of replication, and we propose a model for its amplification. In line with the work of others but following a different approach, we provide support for a potential role of episomal forms in HIV persistence. Our data highlight the biological complexity of HIV replication and the potential of the episomal form to contribute to the persistence of HIV.

**Supplementary Information:**

The online version contains supplementary material available at 10.1186/s12977-025-00658-1.

## Introduction

Reverse transcription and integration are two essential steps in the life cycle of retroviruses. The general model has held that reverse transcription of the viral genome initiates soon after its entry into target cells following the uncoating of the capsid to allow access to deoxynucleotides [1]. Early studies reported the presence of viral DNA in virions and suggested that reverse transcription can initiate before or during the formation of mature viral particles [2–6]. Atomic Force Microscopy (AFM) showed that transcription of the single flexible strand RNA into a more rigid DNA structure increases internal pressure inside the core, thereby initiating capsid disassembly [7]. More recently, studies involving labeling and tracking of viral capsids showed that the process of reverse transcription is completed in the nucleus with disassembly of the intact cores in the proximity of the integration site [8, 9]. In addition to the formation of the provirus, high levels of unintegrated linear and circular viral DNA (1- and 2-LTR episomal forms) are detected in the early phase of infection and decline with time [10]. While integration has long been considered an obligatory step for all retroviruses, the circular DNA forms were considered dead-end byproducts of reverse transcription; side products that do not give rise to infectious progeny [11]. Studies with integrase mutants and reporter viruses confirmed that the episomal forms are capable of some gene expression but are unable to sustain productive infection [12, 13].

This concept would long be debated however, and the search for a role for episomal forms in the life cycle of HIV has been the subject of many studies. Panganiban and Temin [14] were the first to sustain that integration of retrovirus DNA is not an absolute prerequisite for retrovirus replication. They reported that in chicken embryo fibroblasts infected with spleen necrosis virus, “the unintegrated viral DNA (not a rare integrated copy) is used as the transcriptional template during infection by integration deficient virus” although virus production is lower than that observed for integration-competent strains. The path for studying the role of the episomal form was opened, and several laboratories focused their attention on the early events of infection in resting or proliferating cells.

Integration is highly inefficient, and reverse transcribed HIV-1 accumulates as unintegrated DNA in vitro [10] and in vivo in peripheral blood CD4^+^ T cells and monocytes, in lymphoid tissue, and in the brain [15–22]. Longitudinal studies in cohorts of persons living with HIV (PLWH) have shown that episomal DNA is present in untreated as well as in antiretroviral treated individuals [23]. Despite effective antiretroviral treatment (ART), low level HIV viremia persists, but its source is unclear. The following alternative sources have been proposed: ongoing, complete cycles of replication [24–26], a long-lived reservoir(s) of latently infected cells [27, 28], sanctuary sites into which antiretroviral drugs have poor penetration [29, 30], or a combination of these possibilities.

The existence of a cryptic viral reservoir resistant to ART was suggested by studies comparing the env sequences of plasma virus RNA rebounding after treatment interruption with the sequences of episomal and proviral forms present prior to the interruption [31]. A phylogenetic relatedness was observed only with the episomal forms. The episomal forms, present during treatment, but considered unstable and unable to sustain virus replication, were used in these and other studies as markers of new infection.

In support of the idea that the unintegrated forms of HIV DNA could produce infectious virus, Cara et al. [32] transfected HeLa cells with in vitro synthesized 1-LTR and 2-LTR circular HIV DNA and with expression vectors carrying p24 or luciferase reporter genes under the promoter activity of single or two joined LTRs. They demonstrated that these forms are transcriptionally competent in transiently transfected HeLa cells, with the 1-LTR being more efficient than the 2-LTR form, judged by the level of p24 or of luciferase activity. However, the episomal forms continued to be considered dead-end byproducts of aborted infections, only capable of limited transcription [10].

A number of studies in the following years focused on the potential of unintegrated circular DNA to function as a template for the production of a fully replication competent virus. The ability to express viral genes from unintegrated DNA was investigated in macrophages by Kelly et al. [33]. Unlike quiescent CD4^+^ T cells that are resistant to HIV infection due to an early block in reverse transcription, nondividing, metabolically active macrophages can be infected. Compared to proliferating cells, the episomal forms are more stable in these nondividing cells and transcription persists over 30 days of culture.

Using reporter viruses, Gelderblom et al. [34] showed that in cells coinfected with wild type (WT) and integrase (IN)-deficient viruses, late genes can be expressed from unintegrated DNA by the action of Tat and Rev derived from the integrated virus. In addition, they showed that cells coinfected with IN-deficient and WT virus package both genomes in the same virion, and that the unintegrated DNA-derived genome undergoes recombination with the integrated DNA-derived genome during a second round of infection, thus completing its replication cycle through complementation. As Wu [35] commented, a second chance was given for the unintegrated DNA to complete its replicative cycle.

Kantor et al. [36] showed that HIV episomal forms are organized in a nucleosome structure, subject to epigenetic modification as with DNA viruses that do not integrate. They found sustained expression of p24 after addition of histone deacetylase inhibitors in SupT1 cells and monocyte derived macrophages infected with IN-deficient virus, linking the silencing of the unintegrated HIV DNA forms to a repressive chromatin structure. The connection between histone acetylation and transcriptional regulation has been the subject of many studies (contributed to and reviewed by Struhl [37, 38]). The inhibitors used in the Kantor studies were short chain fatty acids, natural products of the gut microbiota [39]. The finding that expression from episomal forms of HIV can be restored once the epigenetic silencing is removed has been addressed in recent studies [40, 41].

The expression of virus from unintegrated DNA in resting CD4^+^ T cells infected with WT or IN-deficient reporter viruses was reported by Trinité et al. [42] using two systems: cells preactivated with anti-CD3/CD28 antibodies, and cells pretreated with IL-4, which renders quiescent lymphocytes permissive to HIV infection without changing the differentiation state [43]. Infecting preactivated, proliferating lymphocytes with a WT reporter virus generates a strong fluorescent signal in the majority of the cells and a dim signal in a minority, with each signal linked to expression of green fluorescent protein (GFP) respectively from proviral or unintegrated DNA. If preactivated cells are infected in the presence of raltegravir or with IN-deficient virus, only GFP + dim cells are observed. Likewise, only GFP + dim cells are observed in similarly infected IL-4 pretreated quiescent cells. However, if these cells are activated at a later time after infection, a consistent population of bright cells is formed, proving that unintegrated DNA can function as a template for de novo production of infectious virus. Complementation experiments demonstrated that the presence of Vpr was required for the full expression of the unintegrated DNA.

The growing interest in the role of the unintegrated DNA in HIV persistence stimulated studies to quantify unintegrated DNA using assays for total unintegrated DNA rather than total DNA or 2-LTR DNA [44] and to develop sensitive, specific technology for the quantification of unintegrated linear DNA [45].

**Fig. 1:**
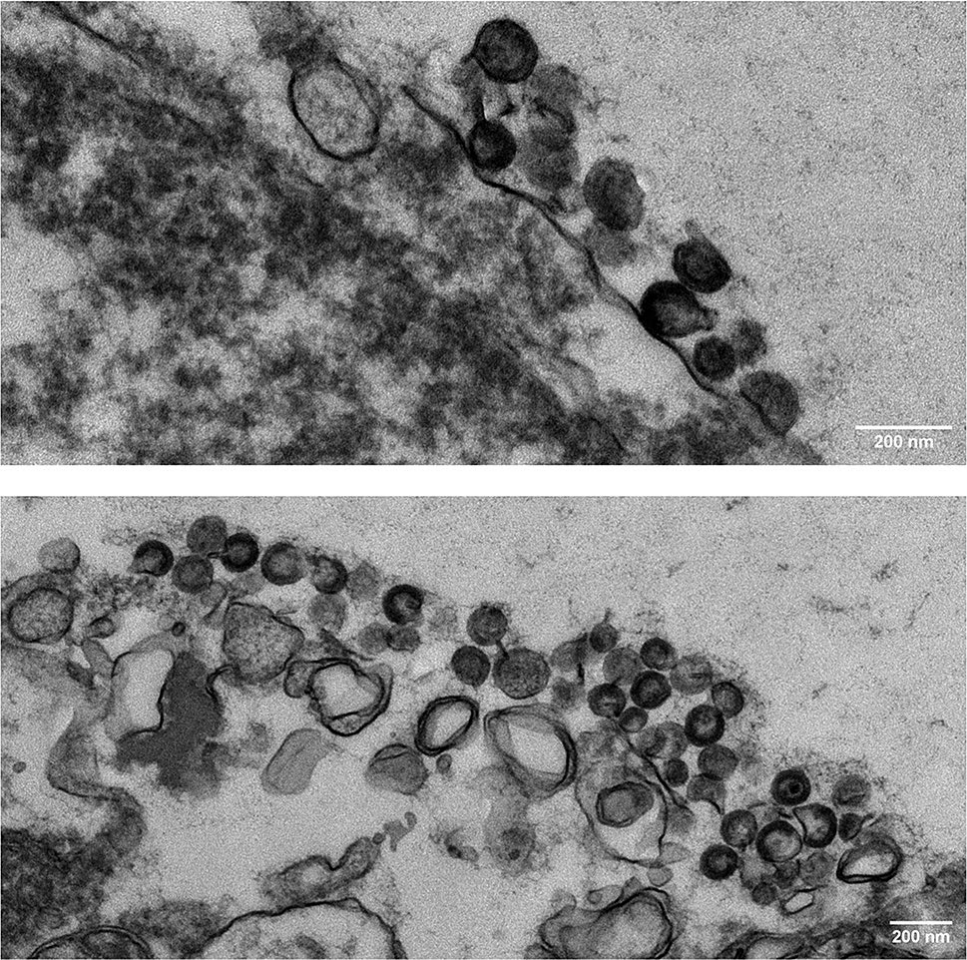
Electron Microscopy analysis of Jurkat cells transfected with 1-LTR_HIV_. Cells were prepared and analyzed using procedure A as described in the Materials and methods for cells growing in suspension. Calibration = 1.083 nm/pixel at 10.0k; magnification = x15.0k

In our study, we further investigated the role of unintegrated DNA in the production of infectious virus focusing on the 1-LTR circle. This is the most abundant of the two episomal forms. It is not easily distinguishable from unintegrated linear DNA, and its contribution cannot be appreciated in the presence of the productive linear form. We demonstrate that viral expression is initiated and completed by a 1-LTR linear form of HIV DNA that upon circularization and amplification in cells lacking CD4 produces infectious virus in the absence of integration. When this linear form is circularized in vitro by T4-ligase-mediated closure, expression of infectious virus is equally obtained. Direct evidence of virus production was provided by electron microscopic analysis both in HeLa and Jurkat cells transfected with episomal HIV DNA (Fig. 1). In addition, we found that the 1-LTR episomal form replicates despite the absence of an origin of replication, and we provide a model for the suggested amplification. This work highlights the biological relevance and contribution of the episomal 1-LTR DNA to the initiation of productive HIV replication that is difficult to distinguish and appreciate in the pool of unintegrated DNA. In line with the work done by others, our study supports the idea that episomal HIV DNA is not just a byproduct of reverse transcription, but that it actively contributes to productive HIV infection.

## Results

### Expression of infectious virus from an episomal form of HIV

**Fig. 2:**
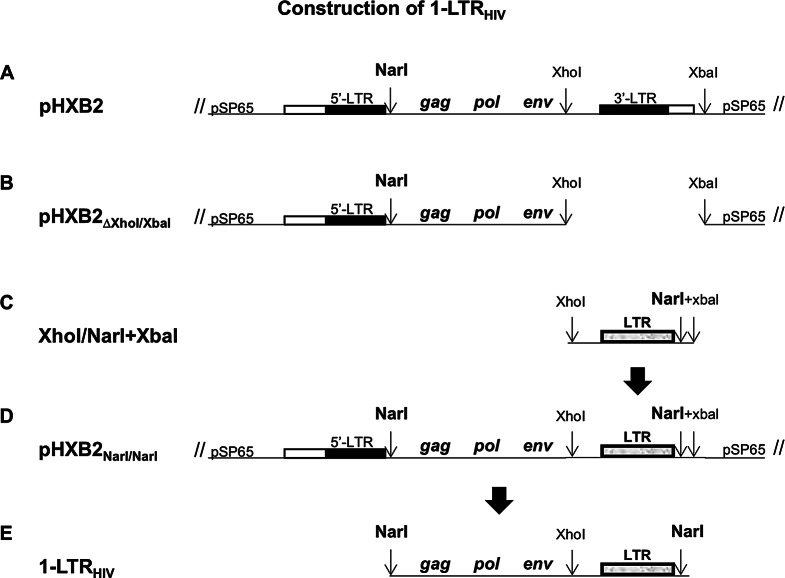
Construction of 1-LTR_HIV_. (**A**) Structure of pHXB2. The empty boxes flanking the LTRs represent cellular sequences. (**B**) Removal of an XhoI/XbaI fragment containing nef sequences, 3’-LTR, and cellular sequences from pHXB2 using the restriction enzyme XhoI that cleaves in the nef sequence and XbaI that cleaves at the end of the cellular sequences. (**C**) PCR amplification of an XhoI/XbaI DNA fragment containing nef sequences and one LTR with the adjacent viral sequence (primer binding site) containing the restriction site NarI. The source material for the PCR was DNA from HXB2 infected Jurkat cells. The primers were designed from unique sequences flanking the 5’- and 3’-LTR of HXB2 (reported in Materials and methods). The PCR product was purified, analyzed on agarose gel, cloned into the pCRII-Topo vector, and transformed into DH5-α competent cells. Ten transformants were isolated and sequenced (Fig. 3). The PCR reaction was carried out using the Invitrogen Platinum Supermix High Fidelity kit. PCR purification was done using the Qiakit PCR purification kit. Agarose gel extraction was done with the Qiagen gel extraction kit. Competent cells and TA ligation kit were obtained from Invitrogen (Waltham, MA) and restriction enzymes from New England Biolabs (Ipswich, MA). (**D**) The recombinant plasmid was digested with XhoI and XbaI and the isolated insert was T4 ligated with pHXB2ΔXhoI/XbaI to obtain pHXB2 NarI/NarI. (**E**) Digestion of this construct with NarI releases the linearized 1-LTR_HIV_ used in this study. Agarose gel extraction and transformation of the competent cells were performed as described above

**Fig. 3:**
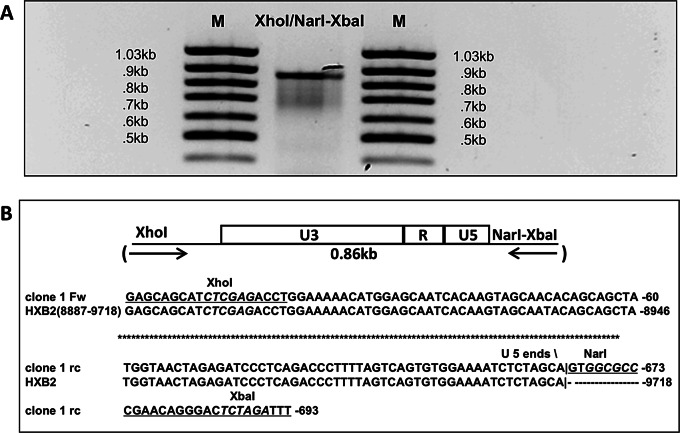
PCR amplification of an HIV DNA fragment containing the long terminal repeat (LTR) of the 1-LTR episomal DNA used for the construction of 1-LTR_HIV_. (**A**) Gel analysis of the XhoI/NarI fragment of 1-LTR episomal DNA present in Jurkat cells infected with HXB2. A DNA fragment containing a single LTR was obtained by PCR amplification of total DNA extracted from a Jurkat cell line (J-20, > 99% CD4^+^) acutely infected with HXB2, using the primers described in Fig. 2. The PCR reaction was carried out using the Platinum Supermix High Fidelity kit (Invitrogen). The PCR product was purified using the Qiakit PCR purification kit, analyzed by gel electrophoresis, cloned into the pCRII-Topo vector, and transformed into DH5-α competent cells (competent cells and TA ligation kit from Invitrogen). The size of the amplicon (0.9 kb) is as expected. (**B**) Sequence analysis of the XhoI/NarI 1-LTR circular DNA fragment amplified from infected Jurkat cells and used to construct 1-LTR_HIV_. Plasmid DNA was extracted from 10 transformants using the Qiagen Plasmid Purification kit. Each plasmid contains an insert of the expected size (0.9 kb) as judged by agarose gel analysis of the EcoRI digests of the respective DNAs. The amplified NarI/XbaI insert was sequenced. The 5’ and 3’ ends of the sequence of one of the isolated amplicons are shown, aligned with the sequence of HXB2. All nucleotide sequences determined in the course of this investigation were obtained from the NCI Core Facility at NIH

To investigate the biological relevance of HIV episomal DNA, we generated a linear HIV DNA that lacks the 5’-LTR, as described in the legend to Fig. 2. This form is contained in and released from a modified molecular clone of HIV (pHXB2) upon digestion with NarI. Briefly, we removed a fragment of pHXB2 containing *nef* sequences, the 3’-LTR, and flanking cellular sequences (XhoI/XbaI, Fig. 2A, B) and replaced it with an analogous XhoI/XbaI fragment amplified from HXB2-infected Jurkat cells. This fragment lacks cellular sequences and, adjacent to the LTR, instead contains the sequences of the primer binding site that include a NarI restriction site (Figs. 2C and 3A and B). The modified pHXB2 thus contains two NarI sites, one normally present in the primer binding site adjacent to the 5’-LTR, and a second one introduced at the 3’-end of the viral DNA upstream of the XbaI site (pHXB2NarI/NarI, Fig. 2D). We next cleaved the pHXB2NarI/NarI with NarI to generate linear genomic viral DNA (1-LTR_HIV_) that lacks the 5’-LTR and has at its two extremities the protruding ends of the NarI restricted primer binding site (Fig. 2E). All experiments described below were performed with the agarose-purified 1-LTR_HIV_ DNA form.

**Fig. 4:**
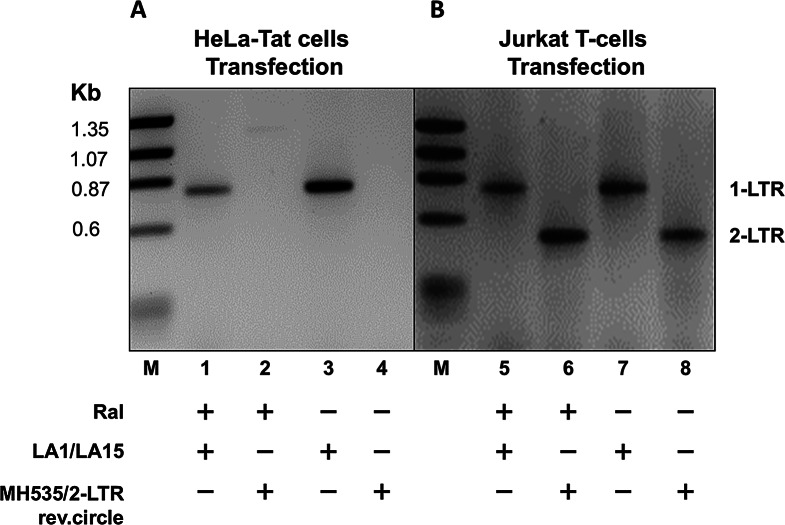
Production of infectious virus from CD4^+^ or CD4^−^ cells transfected with 1-LTR_HIV_. (**A**,** B**) Analysis of 1-LTR or 2-LTR circles generated in cells transfected with 1-LTR_HIV_ in the presence or absence of raltegravir (Ral). Total DNA was extracted from a culture of transfected (**A**) HeLa-tat cells or (**B**) Jurkat cells. Cells were harvested after three days of growth in the presence or absence of 20 nM of raltegravir. The respective sizes of the amplicons are 0.79 kb and 0.5 kb, as expected. The two amplicons were cloned and sequenced (Fig. S1)

HeLa-tat cells (CD4 negative) and Jurkat T cells (CD4 positive) were transfected with the linear 1-LTR_HIV_ grown in the presence and absence of the integrase inhibitor raltegravir [46] and analyzed for the formation of episomal DNA. Transfection of the HeLa-tat cells resulted in the production of only the 1-LTR circular form, as demonstrated by PCR amplification using primers for 1-LTR or 2-LTR circles and gel analysis, and by sequencing of the amplified PCR product (Figs. 4A and B and Fig. S1A). The absence of the 2-LTR form is consistent with a lack of reinfection since HeLa-tat cells do not express the viral receptor (CD4), and excludes the presence, if any, of a complete genomic linear DNA that would generate both circular forms and integrate in the absence of raltegravir. Virus production in these cells is initiated from the circularized form of 1-LTR_HIV_ through transcription initiation and termination signals of the LTR.

Transfection of Jurkat T cells (CD4 positive) resulted in the production of both 1-LTR and 2-LTR circles (Fig. 4B). In the presence of raltegravir, production of both forms is consistent with the circularization of linear 1-LTR_HIV_, virus production from the episomal form, and spreading of the virus in culture. In the absence of drug, virus production initiates from the circularized 1-LTR_HIV_, spreads in culture, and continues to be produced from the episomal and integrated forms.

**Fig. 5:**
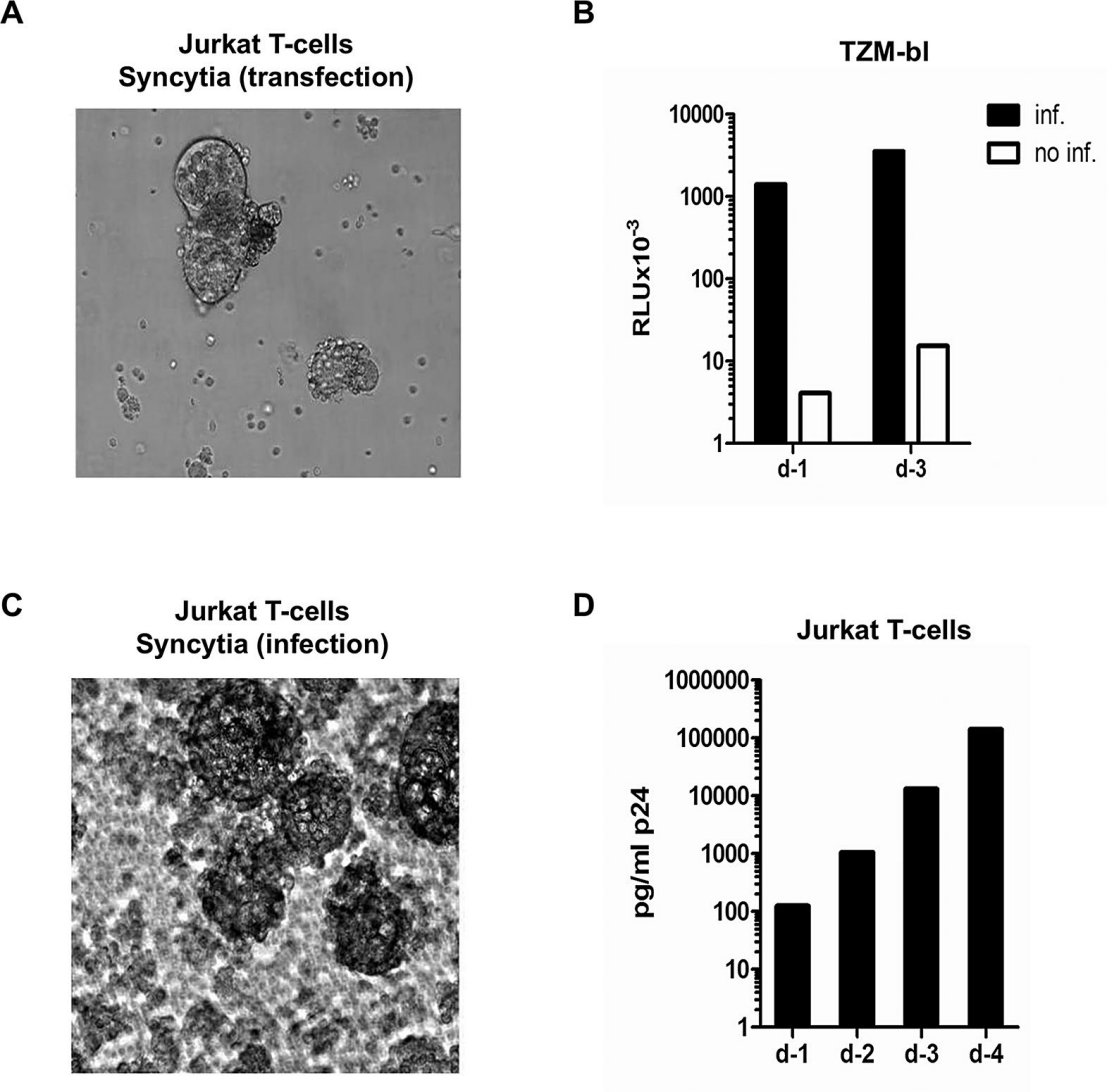
Production of infectious virus from Jurkat cells transfected with 1-LTR_HIV_. (**A**) Syncytia were observed 3 days after transfection. At 4 days, the culture supernatant contained 716 ng/ml of p24. (**B**) Expression of Tat activity in TZM cells incubated with the supernatant of Jurkat cells transfected with 1-LTR_HIV_. TZM cells were infected with 1 ml of culture supernatant containing 316 ng of p24 and processed daily for quantification of luciferase activity. Three days after infection 3.5 × 10^7^ RLU (relative luminescence units) were measured versus 1.5 × 10^4^ in the untreated control. (**C-D**) Infection of Jurkat cells with the supernatant of the transfected Jurkat cells. As is the case with transfected cells, a large number of syncytia was observed as shown. Four days after infection the level of p24 in the culture medium was 141 ng/ml

Transfection of the linear 1-LTR_HIV_ into Jurkat T cells produced HIV able to induce syncytia in the transfected culture (Fig. 5A) and capable of being transmitted to the CD4^+^CCR5^+^ TZM cell line JC52BL-13 [47] as demonstrated by the expression of a Tat-responsive luciferase gene (Fig. 5B). The virus obtained from the supernatant of transfected cells also infected Jurkat T cells as demonstrated by the formation of large and numerous syncytia by day 3 (Fig. 5C) and by the accumulation of p24 in the supernatant (Fig. 5D). Similarly, the supernatant from HeLa cells transfected with 1-LTR_HIV_ as well as the supernatant from 1-LTR_HIV_ transfected primary PBMC were also infectious for Jurkat cells (Fig. S2A, B; Fig. S3A; Table S1).

These data provide evidence that infectious virus is formed from the circularized form of 1-LTR_HIV_ as even in the case of non-integrase dependent integration [48] of the linear form in the proximity of a cellular promotor, only viral proteins, and not infectious virions, could be generated. A genomic viral RNA transcript initiated from a cellular promoter would lack part of the primer binding site and the 5’-U3R region. Consequently, the first strong-stop DNA, necessary to initiate reverse transcription, could not be formed, and the RNA of any assembled virus-like particle would not be reverse transcribed.

To confirm that infectious virus is produced from a 1-LTR circle in our system, we ligated the purified linear 1-LTR_HIV_ DNA in vitro under conditions that favor intramolecular closure and minimize the formation of concatemers [49]. To eliminate linear forms, ligation product was treated with ExoV, a processive, bidirectional exonuclease that digests linear double stranded DNA from both the 5’- and 3’-ends but does not digest circular DNA.

**Fig. 6:**
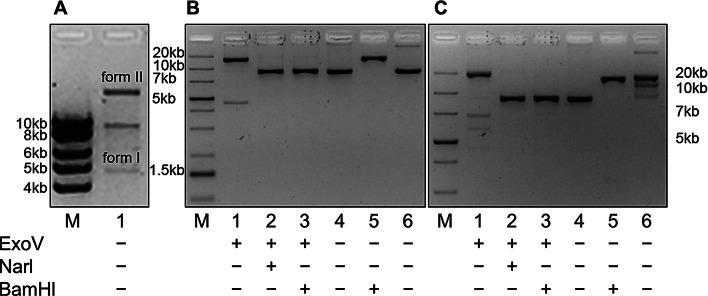
Agarose gel analysis of in vitro circularized 1-LTR_HIV_. (**A**) Different forms of 1-LTR_HIV_ after T4 ligation in vitro: faster moving fragment, form I (supercoil); intermediate fragment, 1-LTR_HIV_; slower moving fragment, form II (open circle). (**B**) Restriction analysis of T4 ligated 1-LTR_HIV_: digestion of circularized 1-LTR_HIV_ with ExoV (lane1), ExoV and NarI (lane 2), ExoV and BamHI (lane 3), and 1-LTR_HIV_ (lane 4); digestion of pHXB2 with BamHI (lane 5) and pHXB2 (lane 6). (**C**) Longer electrophoresis of the gel shown in (**B**), in the presence of additional EtBr. As with the linear form, transfection of Jurkat cells with in vitro circularized 1-LTR_HIV_ produced infectious virus. Syncytia were formed in the culture of the transfected cells, and the culture supernatant induced syncytia in a second culture of Jurkat cells, confirming the infectivity of the virus produced by the in vitro circularized 1-LTR_HIV_ (Fig. S3B)

To demonstrate that only circular forms are generated under these conditions, we analyzed the ligation product by agarose gel electrophoresis before and after ExoV treatment. In the absence of nuclease treatment, three DNA fragments were observed: one of 20-23 kb (form II), a second of 9.1 kb, and a third of 5-6 kb (form I) (Fig. 6A, lane 1). Upon ExoV cleavage, the intermediate 9.1 kb form was eliminated, suggesting that it represents incompletely ligated 1-LTR_HIV_ linear forms, whereas the other two forms were not affected by the exonuclease, confirming their circular nature (Fig. 6B, lane 1). Indeed, cleavage of the ligation product with NarI converted the ExoV resistant forms into a single linear form of 9.1 kb (Fig. 6B, lane 2), consistent with the two circular forms having the same molecular weight but different conformations (forms I and II). The supercoiled nature of the faster moving form was confirmed by the effect of increasing ethidium bromide concentration on its mobility (Fig. 6C, lane 1). A similar effect was observed on the migration of the circular plasmid pHXB2 (Fig. 6B, C, lane 6).

To rule out the possibility that the slower moving form might indeed be a circular dimer, we cleaved the ExoV resistant DNA with BamHI. This enzyme cleaves the HXB2 DNA once in the env gene and, like NarI, would cleave head-to-tail circular dimers into two identical fragments of 9.1 kb. Instead, cleavage of head-to-head/tail-to-tail circular dimers would give two fragments of different molecular weights, easily discernable on agarose gel. As shown in lane 3 of Fig. 6B and C, cleavage with BamHI yielded only the 9.1 kb fragment as in the case of NarI (Fig. 6B and C, lane 2). Cleavage of the topoisomers of pHXB2 with BamHI also yielded a single fragment as demonstrated in lane 5 of Fig. 6B. These results were reproduced in several ligations of 1-LTR_HIV_ carried out under the described conditions, suggesting that concatemer forms, linear or circular, are unlikely to be generated under our conditions for in vitro ligation. The infectivity of virus produced by the in vitro circularized 1-LTR_HIV_ was shown in two systems: first, by using the supernatant of cultures of Jurkat or HeLa-tat cells transfected with the circular form to transmit the virus to Jurkat cells (Fig. S3B, C, E); and second, by indirect cocultivation of peripheral blood lymphocytes (PBL) transfected with the circular form with Jurkat cells using a transwell system (Fig. S4).

### Stability of virus expression from 1-LTR episomal DNA measured by FACS analysis

We next followed the intracellular expression of HIV on a per cell basis from cultures transfected with the linear 1-LTR_HIV_ as an indirect measurement of the stability of its circularized form during cell growth. We used HeLa-tat, a CD4 negative cell line, to avoid the formation of the episomal form by reinfection. The cells were intracellularly stained with a fluorescent antibody against p24, and flow cytometric analysis was performed using Trucount absolute counting tubes.

**Fig. 7:**
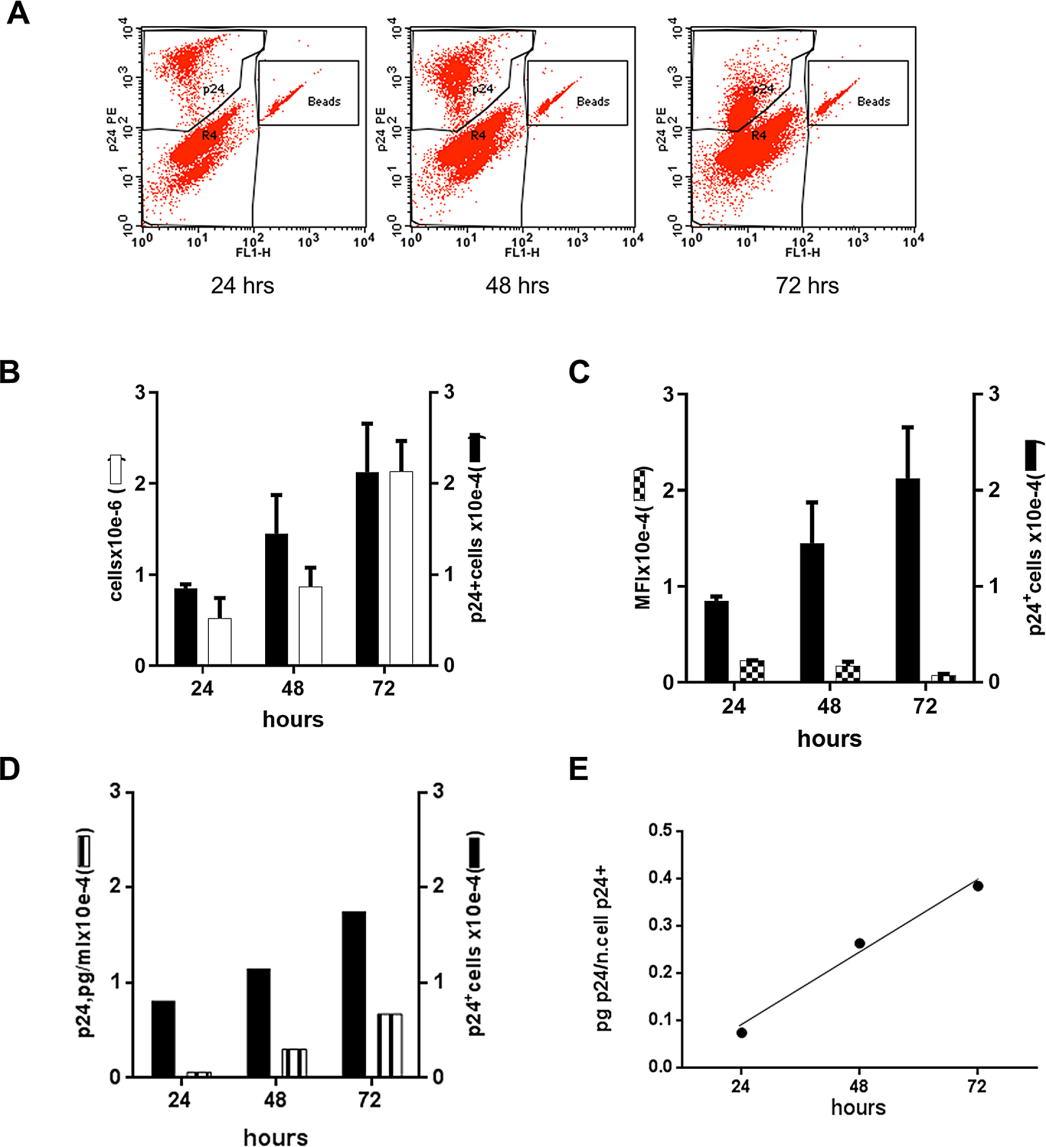
Intracellular and soluble p24 in HeLa-tat cells transfected with 1-LTR_HIV_. (**A**) Flow cytometry analysis of HeLa-tat cells transfected with 1-LTR_HIV_. The absolute number of cells stained intracellularly for p24 was determined by Trucount analysis, as described in the Materials and methods. A representative plot showing untransfected cells and p24^**+**^ cells detected at different timepoints is shown (upper panels). Since Trucount beads fluoresce at multiple wavelengths, data was plotted using two fluorescence detectors (FL-1 and FL-2) to facilitate gating of both Trucount beads and p24^**+**^ cells. (**B**) Growth rate of the transfected and intracellularly stained HeLa-tat cells. The growth rate of the transfected HeLa-tat cells (bulk culture) and that of the subpopulation of p24^+^ cells contained in the bulk culture are represented. As shown, the subpopulation of p24^+^ cells grows at a lower rate than that of the bulk culture. (**C**) Trucount values and mean fluorescence intensity of p24 stained cells. Data are plotted as a function of time. The data reported in (**B**) and (**C**) are the average of two experiments; error bars represent one standard deviation. (**D**,** E**) Intracellular and soluble p24 in HeLa-tat cells transfected with 1-LTR_HIV_. (**D**) The level of p24 in the culture supernatant of the transfected cells and the number of p24^+^ cells, measured at daily intervals, are shown. (**E**) The ratios of soluble p24and the number of p24^+^ cells are plotted as a function of time

**Fig. 8:**
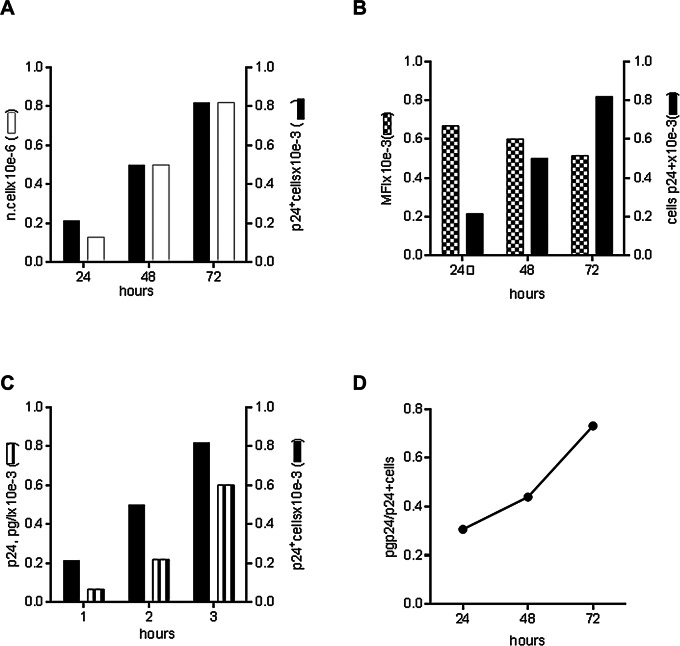
Analysis of soluble and intracellular p24 in HeLa cells transfected with 1-LTR_HIV_. The experimental conditions and the analysis of the data are as described for HeLa-tat cells. (**A**) The growth rate of the transfected HeLa-tat cells (bulk culture) and that of the subpopulation of p24^+^ cells (contained in the bulk culture) are represented. (**B**) Trucount values and median fluorescence intensity of the p24 stained cells are plotted as function of time. (**C**) The level of p24 in the culture supernatant of the transfected cells and the number of p24^+^ cells, measured at daily intervals, are shown. (**D**) The ratio of soluble p24 and the number of p24^+^ cells is plotted as a function of time

Figure 7A and B show that the absolute count of p24^+^ cells/µl of culture increased with time, but their mean fluorescence intensity decreased in the same period (Fig. 7C). Even as their count increased, each cell had a lower expression level of intracellular p24 antigen, suggesting a progressive dilution of the DNA during cell division or a progressive decrease in the expression of HIV DNA. However, both the production of p24 in the supernatant and the ratio of soluble p24 to the number of cells positively stained for intracellular p24 increased with time (Fig. 7D, E). Similar results were also observed with HeLa cells (Fig. 8). Thus, within the timeframe of our experiment, viral production in the bulk culture was linked to the ability of the subpopulation of transfected cells to divide without losing HIV DNA and express p24 efficiently from the unintegrated 1-LTR circle.

### Amplification of 1-LTR episomal DNA measured by real-time PCR

**Fig. 9:**
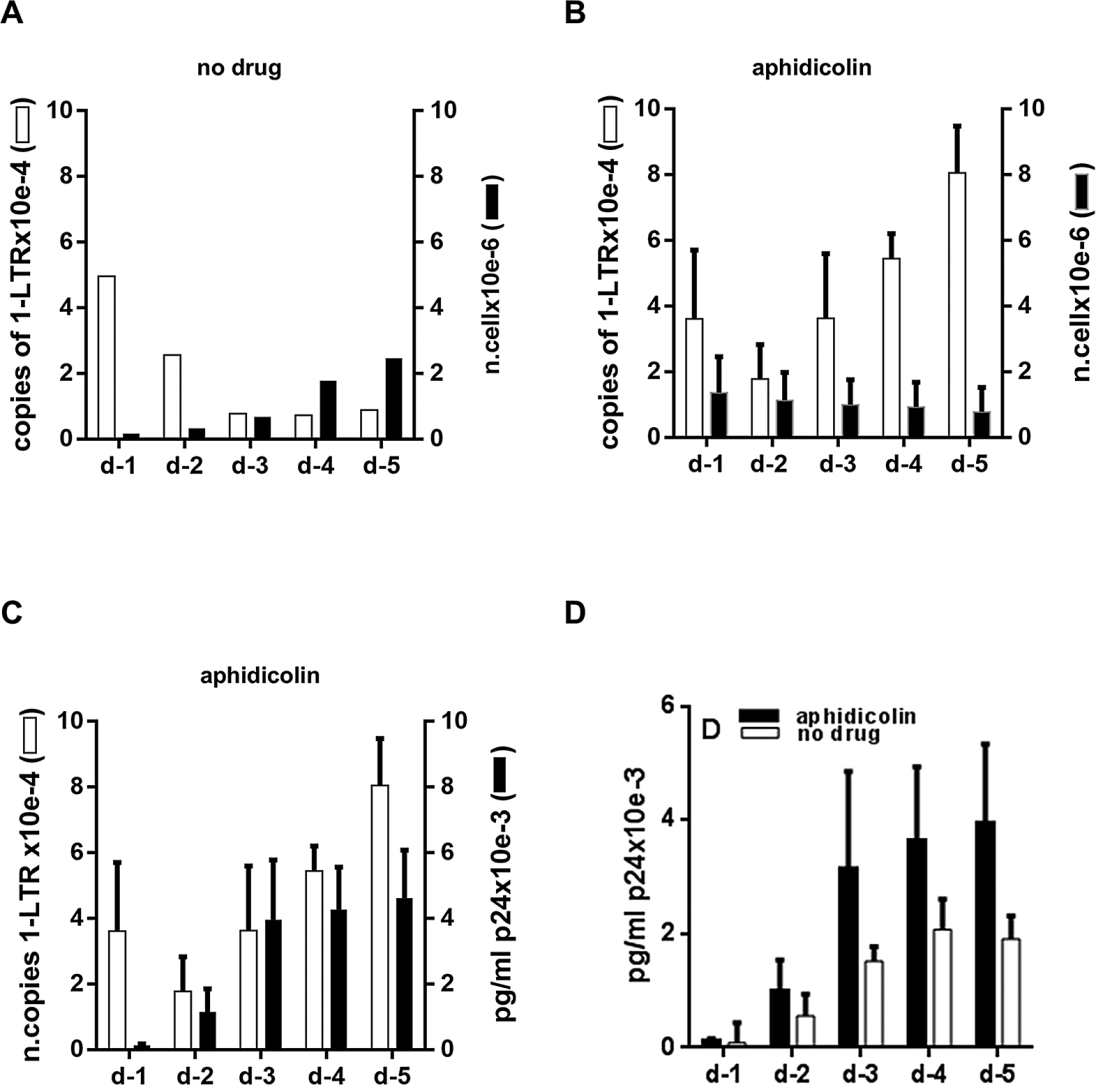
Number of copies of 1-LTR circles in transfected HeLa-tat cells in the presence or absence of aphidicolin. (**A**) HeLa-tat cells transfected with 1-LTR_HIV_ and cultivated in the absence of aphidicolin. The number of copies of 1-LTR circles and the number of cells are plotted as a function of time in culture. The primer set used for the amplification of the 1-LTR circles consisted of: LA1 (forward), LA15 (reverse), and the probe 1-LTR-P. Detected DNA was normalized by measuring the amount of β-globin present in each sample with primers specific for the β-globin gene (see Materials and methods). The initial rapid decline in the number of 1-LTR amplicons is consistent with the disappearance of linear head to tail dimers co-formed by intermolecular ligation of 1-LTR_HIV_. (**B**) HeLa-tat cells transfected with 1-LTR DNA and incubated in the presence of aphidicolin. The number of copies of 1-LTR circles and the number of cells is plotted as a function of time in culture. (**C**) HeLa-tat cells transfected with 1-LTR and cultivated in the presence of aphidicolin. The increase in the number of 1-LTR circles and the amount of p24 detected in the culture supernatants are shown. (**D**) The amount of p24 released in the supernatant of HeLa-tat cells transfected with 1-LTR_HIV_ in the presence or absence of aphidicolin is plotted as function of time in culture. Error bars represent one standard deviation from the average of two experiments, as described

To confirm that 1-LTR_HIV_ is maintained in cells over time and is in a circular form, we performed qPCR analysis on DNA obtained at different timepoints after transfection of cells cultured in the presence or absence of aphidicolin, an inhibitor of DNA polymerase α that blocks progression of the cell cycle through the S phase [50]. We measured the number of copies of the 1-LTR circle on day 1 after transfection (baseline) and at daily intervals thereafter. In the absence of the drug, the number of copies of 1-LTR circles in the HeLa-tat cells declined over time, very likely due to the difference in growth rates between transfected and untransfected cells in the culture (Fig. 9A). In the presence of the drug, in contrast, the number of copies increased with time (Fig. 9B). Accordingly, a progressive accumulation of p24 and copies of circular forms were observed despite the absence of growth of cells treated with the drug (Fig. 9C). The expression of p24 in the presence or absence of drug as a function of time in culture is shown in Fig. 9D. The data obtained with qPCR are consistent with FACS analysis showing that the number of p24^+^ cells detected by intracellular staining of HeLa or HeLa-tat cells transfected with 1-LTR DNA and the level of soluble p24 increase with time in culture.

## Discussion

Provirus integration into the host chromosome is, as for other retroviruses, recognized as an essential step in the replication of HIV, whereas the circular forms have long been considered incapable of a productive life. However, the interest in defining the role of episomal viral DNA in the life cycle of HIV never ceased. Fueling this interest were studies showing that the inefficiency of integration [7, 8] resulted in the accumulation of unintegrated DNA in vitro [10] and in vivo, in treated and in untreated persons with either high or low viral loads [20, 51–53]. In addition, a large percentage of defective provirus is found in persons living with HIV [54, 55]. With time on antiretroviral treatment, the ratio of unintegrated to integrated HIV decreases, but unintegrated HIV DNA (as measured by 2-LTR forms) is still detected in a subset of individuals on long-term treatment [56].

Over the years, efforts to define the role that the episomal forms have in the life cycle of HIV continued with various approaches. Work from independent laboratories provided evidence that a complete virus can be produced from unintegrated DNA. Kantor et al. [36] linked the downregulation of transcription of unintegrated HIV DNA to a repressive chromatin structure subject to epigenetic regulation. The addition of histone deacetylase inhibitors increased gene expression and replication of nonintegrating HIV-1.

The mechanisms inducing repressive epigenetic modifications of transcription have been elucidated. Unintegrated DNA becomes loaded with core and linker histones and is subject to modifications regulating the accessibility of DNA to the transcriptional machinery [58]. The magnitude of silencing is not identical in all cell types, and the silencing of the DNA of different retroviruses are indeed mechanistically distinct [40, 42, 57–59]. Work in the Cullen laboratory [40] showed that the expression of Tax, the transcription factor of the human T-cell leukemia virus 1 (HTLV-1), reverses the epigenetic silencing of unintegrated DNA in cells infected with IN-deficient HIV, allowing the spread of virus infection due to active transcription from episomal DNA templates. They speculate that, since Tax can rescue the replication of IN-deficient HIV-1, integrase inhibitors may be less effective in the treatment of dually HTLV-1/HIV-1 infected individuals.

Trinité et al. [42] have reported conditions for expressing virus from episomal DNA. They found that by activating IL-4-pretreated, resting T cells several days after infection with IN-deficient HIV, de novovirus was generated from unintegrated DNA without the help of integrated provirus. If, however, infection occurred after activation, only transient and reduced levels of gene expression were obtained from the unintegrated DNA.

The consensus that emerges from these studies is that under defined experimental conditions unintegrated DNA is capable of active transcription and can potentially participate in the synthesis of new virions. In line with these studies, but using a different approach, we also provide support for the idea that fully infectious virus can be produced from unintegrated viral DNA. We dissected one aspect of the complex replication cycle of HIV by focusing on the smaller and more abundant of the two circular forms, the 1-LTR [60–62]. Unlike the 2-LTR circle that is formed via the cellular non-homologous end joining pathway, the 1-LTR circle is formed by the cellular homologous recombination process and also is a direct product of reverse transcription. Unlike 2-LTR, the 1-LTR form cannot be easily distinguished from linear unintegrated DNA [63], and its contribution to the expression of viral proteins by unintegrated DNA cannot be appreciated in the presence of the other forms (linear and 2-LTR).

Retroviral DNAs cloned into plasmid expression vectors are routinely used to initiate virus spread in transfection experiments. In these plasmids the initiation and termination signals for transcription are separated at the 5’ and 3’ ends of the viral genome as in the case of a provirus, and the LTRs are flanked by cellular sequences defined by the restriction sites utilized for the isolation of the viral DNA. Expression of infectious virus from naked 1-LTR or 2-LTR HIV DNA has not been shown.

We investigated the ability of the 1-LTR circular form to produce virus using a variety of cell types and conditions. In the process, we observed amplification of this form in the absence of integration. First, we derived a linear form of HIV DNA from a modified molecular clone of HIV corresponding to a 1-LTR circle opened with NarI (1-LTR_HIV_). This restriction enzyme cuts inside the sequence of the primer binding site (pbs), leaving the two protruding ends with two complementary base pairs that can re-anneal to form the 1-LTR circle. The linear 1-LTR_HIV_ that we utilized in this study is transcriptionally incompetent as it lacks the 5’-LTR, but it is rendered competent upon circularization. We introduced by nucleofection the linear 1-LTR_HIV_ into cells lacking CD4, relying on cellular ligases for circularization. In the timeframe of our experiments, we observed accumulation of p24 and infectious virus in the supernatant of these cells. Transmission of virus produced in primary or cultivated cells transfected with the linear 1-LTR_HIV_ was highly reproducible. The production of infectious virus from the circular form was confirmed with in vitro circularized 1-LTR_HIV_, under conditions that excluded the formation of concatemers. The low concentration of circular forms measured by RT-qPCR in our study is very likely a consequence of the instability of the linear DNA, thus reducing the pool of intact molecules of 1-LTR_HIV_ that can be ligated by cellular enzymes.

The amplification of 1-LTR circles as suggested by the qPCR analysis is puzzling since no origin of replication has been found in episomal HIV DNA. Others have used aphidicolin to demonstrate the association of cell cycle arrest and accumulation of unintegrated DNA in cell lines infected with HIV [64]. Bell et al. [65] described interchromosomal clusters of unintegrated DNA at the single cell level in cells infected with HIV. According to the authors, this accumulation could not be interpreted as being due to viral DNA amplification by cellular DNA polymerases, as with DNA viruses, because it was not inhibited when infection occurred in the presence of aphidicolin. It was instead inhibited by AZT, an inhibitor of the reverse transcriptase, and therefore interpreted as a consequence of multiple infections of the same cell. In the HeLa-tat cells utilized in our system, the observed amplification of the 1-LTR circle could not be attributed to reverse transcription following reinfection because these cells do not express the receptors necessary for virus entry, and therefore the produced virus cannot spread and generate additional circular forms. If the observed amplification were the result of continuous intracellular reverse transcription, 2-LTR circles would also have been produced and easily detected following transfection of cells lacking CD4 with the 1-LTR circles (Fig. 4).

We hypothesize that the amplification we observed may have resulted from the DNA-dependent activity of the reverse transcriptase, and we propose a mechanism for the synthesis of the positive and negative strands. In the process of reverse transcription, the polypurine tract (ppt) and primer binding sequence (pbs) can be viewed as the origin of replication for the synthesis of viral DNA. In an analogous way, binding of ppt (generated by RNase H activity) and of the tRNA to their respective complementary sequences on 1-LTR circular DNA could prime the synthesis of the (+) and (-) DNA strands, thus providing a mechanism for the observed amplification. For the generation of ppt a first round of intracellular reverse transcription of genomic HIV RNA is required. The presence of transcriptionally competent 1-LTR circles in our system satisfies this condition. Ppt is released intact by the RNase H activity of the RT as shown by early [66, 67] and more recent studies [68]. It is conceivable that it would be recyclable and thus available for the synthesis of (+) DNA strands over the 1-LTR circles; the binding of the linear ppt to the homologous sequences on the circular DNA should not be impeded by thermodynamic barriers given the flexibility of this short oligo-RNA.

As for the tRNA primer, the elongation of the (+) DNA strand, primed by ppt, over the (-) DNA strand, is extended over the 3’-end of the tRNALys that is linked to the 5’-end of the (-) DNA strand; the extension is stopped by a steric interference caused by the presence of a N^1^-methyl-adenosine at position 58 of the tRNALys (a modification that occurs in most eukaryotic tRNAs [69]). Thus, the reverse transcription of this molecule is limited to eighteen 3’-terminal nucleotides (nt) corresponding to the pbs, by the modified adenine. The stop enables the RNase H, the active site of which is 18 nt distant from the polymerase site of the RT, to cleave the tRNALys between the terminal ribonucleotide A and ribonucleotide C. If the tRNA were fully transcribed the reverse transcription of HIV RNA would be aborted since the elongation of the plus strong stop DNA translocated to the 3’-end of the retrotranscribed genomic RNA would be prevented by the linked DNA copy of the tRNA [70]. Unlike the RNase H of MuLV that releases the tRNA primer intact, the RNase activity of the HIV RT excises the 3’-terminal adenosine from the tRNALys during its removal (reviewed in [71]).

Even if tRNALys were destroyed during retrotranscription of the genomic HIV RNA, more tRNALys primers are available in the transfected HeLa cells, demonstrated by the production of infectious virus. Twenty to twenty-five tRNALys molecules together with an equal number of tRNALys synthetase molecules have been reported per HIV particle. Of these, approximately 8 molecules of tRNALys3/virion have been estimated [72, 73] as potential primers for the amplification of episomal DNA during infection. Lysyl-tRNA synthetase (LysRS) is normally sequestered in a multi-aminoacyl-tRNA synthetase complex (MSC) localized in the cytoplasm. In HIV infected cells, LysRS is redirected from the MSC to the nucleus [73]. Mature tRNAs are constitutively imported from the cytoplasm to the nucleus by a tRNA retrograde process [74–76]. Once reaching the nucleus, the conditions for amplification of the circularized linear DNA, transfected in our system, are in place.

Recent studies modifying the current understanding of post-entry replication events provide support for our hypothesis. These studies have demonstrated that reverse transcription does not require disassembly of the viral capsid soon after entry into target cells in order to access deoxynucleotides: transcription proceeds inside the intact core and is completed in the nucleus before uncoating occurs [7, 8]. Thus, the presence of the reverse transcriptase in the nucleus and the formation of transcriptionally competent episomal forms satisfy the conditions for the generation of ppt and the amplification of the circular forms in the model we propose. This also provides an alternative explanation to that suggested by Bell et al. (multiple rounds of re-infection of the same cell [65]), for the accumulation of episomal DNA that occurs in the early phase of infection. Amplification of an episomal form would provide more templates for the formation of virions and spread to neighboring cells, particularly in the case of a low multiplicity of infection.

Epigenetic silencing of viral DNA may play a role in the reduction of viral load in infected individuals following ART [77]. Despite the existence of this mechanism, a limited level of transcription is observed in persons under antiretroviral treatment [78]. ART, however, has no direct effect on transcription. Since the mechanism of histone modification underlying the epigenetic silencing of RNA transcription and the magnitude of silencing are not identical in all cells [60], it seems reasonable to hypothesize that in some cell types the episomal forms may respond differently to epigenetic regulators of transcription and allow a low level of expression that could fuel virus rebound upon interruption of ART. In addition, expression from episomal forms would be resistant to the effect of currently used antiretroviral drugs that target the reverse transcriptase or integrase phases of the virus life cycle.

By focusing on the smaller transcriptionally efficient 1-LTR circles, we provide evidence that infectious virus is produced in the absence of integration, despite the existence of silencing epigenetic mechanisms. Given the inefficiency of the integration process, the evidence of a large number of deleted proviruses, and our results showing that there is no inherent restriction for the expression of infectious virus from an episomal DNA, it is reasonable to suggest that the episomal forms are an integral component of the reservoir. In conclusion, we propose that HIV DNA episomal forms are biologically relevant and contribute to HIV persistence. Our data offer new insight into the life cycle of HIV and highlight a mechanism for virus persistence that should be considered in pursuit of a strategy to eradicate HIV.

## Materials and methods

### Reagents

HeLa, HeLa-tat, TZM-bl cells, and raltegravir (MK-0518) were obtained from the NIH Research and Reference Reagent Program. Jurkat cells were obtained from the American Type Culture Collection (ATCC). Competent cells for transformation were obtained from Invitrogen. Aphidicolin and enzymes were purchased from Sigma-Aldrich.

### 1-LTR_HIV_ preparation

One mg of plasmid pHXB2 was digested with 5000U of restriction enzyme Narl for 4 hrs, generating two fragments of 9.1 kb (insert) and 5.5 kb (vector). After 20’ of heat inactivation, NaCl at 100 mM final concentration and 1500U Bgll were added to the mixture which was further incubated for 4 h to restrict the size of the vector. The 9.1 kb fragment was separated by agarose gel electrophoresis.

### Transfection

(1) HeLa-tat cells were cultivated in DMEM medium enriched with 10% FCS, 1% pen/strep, and L-glutamine and transfected by nucleofection using the Amaxa (Lonza, Walkersville, MD) nucleofector Kit R for HeLa cells, following the protocol provided by the manufacturer. Before nucleofection, cells were briefly trypsinized, washed with phosphate buffer saline (PBS), and resuspended in PBS at 1 × 10^6^ cells/ml. Aliquots of 1 × 10^6^ cells were centrifuged for 10 min at room temperature at 200xg, resuspended in 100 µl nucleofection solution containing 2 µg of linear 1-LTR_HIV_ unless otherwise indicated, and electroporated using the program for high expression efficiency. The electroporated samples were individually diluted in 2 ml of prewarmed medium, pooled, and distributed equally in a 6 well plate. (2) Jurkat cells were cultivated in RPMI medium containing 10% FCS, 1% pen/strep, and additional L-glutamine and transfected using the Amaxa nucleofector Kit V for Jurkat cells using the program provided by the manufacturer. Aliquots of 1 × 10^6^ cells were transfected with 2 µg of 1-LTR_HIV_ unless otherwise indicated. The electroporated samples were treated as described for the electroporated HeLa-tat cells.

### Infection

Samples of 2 × 10^6^ Jurkat cells were incubated for 2 h in a CO_2_ incubator in 1 ml of culture supernatant from the transfected Jurkat cells and gently shaken every 15 min. Thereafter, the supernatants were removed, cells washed 3 times in 10 ml of PBS and resuspended in 5 ml of RPMI medium. Supernatants were removed daily and analyzed for p24 using the Retro-Tek HIV-1 p24 Antigen ELISA kit (ZeptoMetrix, Buffalo NY).

### Luciferase assay

TZM-bl is a HeLa cell line engineered to express the cellular receptor CD4 and co-receptor CCR5. The cell line contains an integrated reporter gene for firefly luciferase under the control of the HIV LTR [47]. Samples of 2 × 10^5^ TZM cells from an exponentially growing culture were seeded in duplicate in a 6 well plate in 2 ml of DMEM containing 10% FCS and 1% pen/strep. Upon cell adhesion, medium was removed, and the cells incubated for 5 hrs with 1 ml of a culture supernatant from the transfected Jurkat culture. Thereafter, the supernatant was removed, cells washed 3 times with PBS, and incubated at 37^o^C in 2 ml of fresh medium. At the indicated times cells were lysed and the luciferase activity determined using 10 µl of lysate and the kit and protocol of the Promega Luciferase Assay System.

### Ligation

Ligation of 1-LTR_HIV_ was carried out at 16^o^C overnight using 5,000 U/ml of T4 ligase at 20 U/ng DNA. Thereafter, 50 U/ml of ExoV was added and the mixture further incubated for 1 h at 37^o^C to eliminate any form of linear concatemer. The assay mixture was then concentrated, desalted, and further purified using the Qiagen (Carlsbad, CA) PCR purification kit. The eluate was precipitated with EtOH and resuspended in H_2_O.

### Intracellular HIV-1 core antigen (p24) expression measured by flow cytometry

HeLa or HeLa-tat cells transfected with 1-LTR viral DNA were detached with trypsin-EDTA (Life Technologies, Carlsbad, CA), washed with D-PBS (Life Technologies), and resuspended in 200 µl of Cytofix/Cytoperm solution (BD Biosciences, San Jose, CA). The cells were incubated for 30 min at room temperature followed by the addition of 10 ml of D-PBS and stored at 4 °C for up to 3 days prior to staining for flow cytometric analysis. For p24 staining with absolute count analysis, the cells were centrifuged at 400xg for 10 min, the supernatant decanted, and 100 µl of 1X Perm/Wash (BD Biosciences) added. PE-conjugated HIV-1 core antigen (p24) antibody (Clone KC57, Beckman Coulter, Pasadena, CA) was added (10 µl per tube) and the samples incubated at room temperature for 30 min in the dark. The cells were washed with 1 ml of Perm/Wash and all of the supernatant removed. The samples were resuspended in 100 µl of D-PBS and transferred to Trucount tubes (BD Biosciences). The volume was adjusted to exactly 500 µl with D-PBS, and the samples were acquired on a FACSCalibur flow cytometer equipped with 488 nm and 633 nm lasers and CellQUEST Pro software (BD Biosciences). Because of the large size difference between HeLa and HeLa-tat cells and Trucount beads, log amplification was used on the light scatter channels to visualize both populations. Since Trucount beads fluoresce at multiple wavelengths, data was plotted using two fluorescence detectors (FL-1 which has a 530/30 nm bandpass filter and FL-2 for the PE-p24 staining with a bandpass filter of 585/42 nm) to facilitate gating of both the Trucount beads and p24^+^ cells. For absolute count analysis, sample acquisition continued until 10,000 Trucount beads were acquired with the number of cells acquired in the same amount of time, and absolute counts were determined using the following formula:

$$\eqalign{& \>\left( {{{{\rm{No}}.\>{\rm{of}}\>{\rm{events}}\>{\rm{in}}\>{\rm{region}}\>{\rm{containing}}\>{\rm{cells}}} \over {{\rm{No}}.\>{\rm{of}}\>{\rm{events}}\>{\rm{in}}\>{\rm{Trucount}}\>{\rm{bead}}\>{\rm{region}}}}} \right) \times \> \cr & \,\,\,\,\,\,\,\,\,\,\,\,\,\,\,\,\,\,\,\,\,\,\,\,\,\,\,\,\,\,\,\,\,\,\,\,\,\,\left( {{{{\rm{No}}.\>{\rm{of}}\>{\rm{beads}}\>{\rm{per}}\>{\rm{test}}} \over {{\rm{Test}}\>{\rm{volume}}}}} \right) \cr & \,\,\,\,\,\,\,\,\,\,\,\,\,\,\,\,\,\,\,\,\,\,\,\,\,\,\,\,\,\,\,\,\,\,\, = {{{\rm{absolute}}\>{\rm{count}}\>{\rm{of}}\>{\rm{cells}}} \over {\mu \>{\rm{l}}}} \cr} $$

### Aphidicolin

In experiments testing the effect of aphidicolin on the stability of the 1-LTR circles, aliquots of 1 × 10^6^ HeLa-tat cells were transfected with 2 µg of 1-LTR_HIV_ and seeded as indicated above. One million cells were added to the wells to be treated with drug while 0.5 × 10^5^ cells were added to the wells of the control plates to allow longer incubation times. Twenty-four hours later (d-1) cells and supernatants were removed from one well for baseline measurements, and aphidicolin (at 10 µg/ml), or DMSO as control, was added to the other wells. Thereafter, cells and supernatants were removed at 24 h intervals (d-2/d-5) for cell counts, p24 analysis, and quantification of 1-LTR circles by real-time PCR. Because the number of cells seeded in the wells of the control plate was one-half the number of cells seeded in the presence of drug, a correction was introduced when reporting the levels of p24 by multiplying the values measured in the control culture by two.

### Raltegravir

HeLa-tat or Jurkat cells, transfected as described above, were resuspended and grown in medium containing 20 nM of raltegravir. Cells and supernatants were collected daily and frozen for analysis.

### RT-qPCR quantification of 1-LTR circles

Total DNA was isolated from samples of HeLa-tat cells transfected with1-LTR_HIV_ and cultured in the presence or absence of aphidicolin. DNA was extracted by using the Qiagen DNeasy Blood and Tissue kit and subjected to qPCR assay. The forward and reverse primers used for the amplification of the 1-LTR were, respectively, 5’-GCGCTTCAGCAAGCCGAGTCCT-3’(LA1), 5’-CACACCTCAGGTACCTTTAAGA-3’(LA15), and the probe was {6-FAM} AGTG GCGAGCCCTCAGATCCTGC {Tamra-Q} (1-LTR-P). The forward and reverse primers for the β-globin gene were: 5’-CTATTGGTCTCCTTAAACCTG-3’ and 5’-CGGCTGTCATCACTTAGACC-3’, and the probe was {6FAM} CTCAGGAGTCAGATGCACCA {Tamra-Q}.

Control reactions were performed with no DNA template added. One step qPCR assays were performed in triplicate using One-step RT-PCR kit (ThermoFisher Scientific, Foster City, CA) in 25 µl volumes, containing 500 ng total DNA, 12.5 µM of each primer, and 5 µM of probe. To make a standard curve for the copy number quantification, plasmid containing the housekeeping globin gene and the 1-LTR were generated. For each run, a standard curve was generated from triplicate samples of dilutions of the purified DNA ranging from 10^7^ to 1 × 10^1^ nominal copy equivalents/reaction. Copy number of test samples was determined from the control standard regression curve by interpolation of the experimentally determined C_T_ value.

### Primers utilized for the construction of 1-LTR_HIV_

1) Forward primer: 5’-GAGCAGCA TCTCGAGACCTG-3’, nt 8887►nt 8906, containing an XhoI restriction site; 2) Reverse primer: 5’-AAATCTAGAGTCCCTGTTCGGGCGCCAC-3’, nt 653►nt635, containing a NarI restriction site and an additional XbaI site for cloning purposes). The following cycling parameters were used: 2 min at 94^o^ C (initial denaturation); 30 s at 94^o^ C (denaturation), 30 s at 60^o^ C (annealing); 1 min at 72^o^ C (extension); 40 cycles; 10 min at 72^o^ C (final extension).

### Electron microscopic analyses

A) Jurkat cells infected with HIV were collected by centrifugation and fixed in 2% paraformaldehyde, 2.5% glutaraldehyde in 0.1 M PIPES, pH7.4, for one hour and stored overnight at 4^o^C. After fixation, cells were quenched in 50mM glycine and 0.1 M PIPES for 15 min, washed and enrobed in 2.5% low melting point agarose. Agarose containing cells were trimmed into ~ 1mm^3^ cubes, post fixed with 1% osmium tetroxide, 0.75% potassium ferrocyanide in 0.1 M PIPES at room temperature for one hour, washed with water three times, followed by en bloc staining with 1% uranyl acetate in water for one hour. Specimens were then washed with water three times and dehydrated using 30%, 50%, 70%, 90% and 100% ethanol in series, 10 min each. This was followed by two more changes of 100% ethanol and two more changes of 100% acetone before infiltrated with Epoxy resin (Polybed812, Warrington, PA) following manufacturer’s recommendation. Specimens were embedded in Epoxy resin and polymerized at 60^o^C overnight. Resin blocks containing cells were trimmed and sectioned using a Leica UC6 ultramicrotome (Leica Microsystems, Inc., Bannockburn, IL). Silver colored (~ 70 nm) ultrathin sections were collected onto 200 mesh Cu TEM grids, counterstained with uranyl acetate and lead, and examined in a Hitachi 7800 transmission electron microscope operated at 80 kV (Fig. 1). Digital images were acquired using an AMT bottom mount camera (Nanosprint12, Advanced Microscopy Techniques, MA; Fig. 1).
B) Aliquots of 1 × 10^5^ HeLa cells transfected with 1-LTR_HIV_ were seeded on glass coverslips in a 24-well plate, briefly spun at 1000 rpm, and incubated in DMEM with 10% FBS. After overnight growth, the cells were washed with 0.1 M PIPES buffer at pH7.4 and fixed in 2.5% paraformaldehyde and 2% glutaraldehyde in PIPES buffer for 30 min at room temperature, followed by 30 min at 4 °C. The fixed cells were washed thoroughly with buffer to remove residual fixative before processing for TEM embedding as described for Jurkat cells. Electron microscopic sample preparation and image analysis were performed by the Electron Microscopy Laboratory (EML) at the Frederick National Laboratory for Cancer Research, NCI, NIH (Fig. S5).

## Electronic supplementary material

Below is the link to the electronic supplementary material.

Supplementary Material 1

Supplementary Material 2

## Data Availability

Data is provided within the manuscript or supplementary information files.
