# Supplementary material for: Expression of HIV from a 1-LTR circular DNA in the absence of integration
Source: Retrovirology. 2025 Mar 17;22:2. doi: 10.1186/s12977-025-00658-1 (PMC11912779; doi:10.1186/s12977-025-00658-1)
Supplement: Supplementary file 1 — Supplementary Material 1 [file 12977_2025_658_MOESM1_ESM.pdf]

Fig. S1. Sequence analysis of transfected circular DNAs. (A) Sequence analysis of the 1-LTR circle detected in HeLa-tat cells transfected with 1-LTR_{HIV}. For the detection of the 1-LTR circles, the primers LA-1 and LA-15 were used. Ten amplicons were isolated and sequenced. The sequence of the primer binding site containing the correct NarI restriction site formed upon the *in vivo* ligation of 1-LTR_{HIV} is shown for one of the isolated amplicons. The sequences of LA-1 and LA-15 are highlighted. (B) Sequence analysis of 2-LTR circles detected in transfected Jurkat cells. The following primers were used for the detection of the 2-LTR circles: forward primer, MH535 (5'-AACTAGGGAACCCACTGCTTAAG-3', (nt 9585→9607); reverse primer, 2-LTR reverse circle (R) 5'-CAGCGGAAAGTCCCTTGTAG-3', (nt 363→344). The following cycling parameters were used: 2 min at 94ºC (1 cycle initial denaturation); 30 sec at 94ºC (denaturation), 30 sec at 60ºC (annealing), 1 min at 72ºC (extension), 35 cycles; 7 min at 72ºC (final extension). The sizes of the respective amplicons were 0.79kb (1-LTR) and 0.5kb (2-LTR), as expected.

Fig. S2. Transmission of infectious virus from HeLa cells transfected with 1-LTR_{HIV} (HeLa/1-LTR_{HIV}) to Jurkat cells. Virus transmission was demonstrated by (A) co-cultivating HeLa/1-LTR_{HIV} cells with Jurkat cells and using the co-culture supernatant to infect a second culture of Jurkat cells, or (B) cultivating HeLa/1-LTR_{HIV} cells and using the culture supernatant to infect Jurkat cells. HeLa/1-LTR_{HIV} cells were seeded at $3.75x10^5$ cells/well in 2 mls of DMEM +10% FCS in four wells of a six well plate. Twenty-four hours later, medium was removed, $1x10^6$ Jurkat cells in 4 ml of RPMI +10% FBS were added to 2 wells, and RPMI + 10% FCS alone was added to the other two wells. Four days later, cells were counted and the supernatant frozen for p24 analysis. The co-culture released 66 ng p24 per well (A), and HeLa/1-LTR_{HIV} cells alone released 0.4 ng p24 per well (B). $2x10^6$ Jurkat cells were infected with 1 ml of co-culture supernatant containing 16 ng p24 (A) or with 1 ml of supernatant from the HeLa/1-LTR culture containing 148 pg p24. After infection, cells were counted, washed three times with PBS, and resuspended in 6 ml RPMI + 10% FCS. Only the cells infected with the co-culture supernatant developed syncytia 4 days after infection. At this time, soluble p24 was measured, cells counted, washed three times with PBS, and transferred into 20 ml RPMI for expansion. During the subsequent expansion, syncytia also developed

in the culture of cells that had been infected with the HeLa/1-LTR$_{HIV}$ supernatant, and high levels of p24 were produced as shown (160 ng/ml). Data are summarized in Table S1.

Fig. S3. Production of infectious virus from human PBL transfected with 1-LTR$_{HIV}$, and from HeLa-tat or Jurkat cells transfected with linear or *in vitro* circularized 1-LTR$_{HIV}$. (A) Virus transmission via indirect cocultivation of Jurkat cells from human PBL transfected with 1-LTR$_{HIV}$. Two cultures of 5x10^6 PBL were stimulated with PHA for 24 hrs. Thereafter, the medium was changed, and cells were washed with PBS and incubated for 4 days in RPMI containing 100U IL-2 per ml. The stimulated cells were transfected with 2 µg of 1-LTR$_{HIV}$ according to the Amaxa protocol and seeded into two wells of a BD Falcon companion tissue culture plate in IL-2-free RPMI (see Fig S4). A cell culture insert was placed into each of the two wells. 0.5x10^6 Jurkat cells were added into one of the two inserts and cultured for 5 days. PBL and supernatants were removed and frozen, Jurkat cells were washed, resuspended in RPMI, and allowed to expand for one week, at the end of which time numerous syncytia were present as documented in the figure. (B) Virus transmission from Jurkat cells transfected with *in vitro* circularized 1-LTR$_{HIV}$. Six hundred ng of ligated DNA were used to transfect 1x10^6 Jurkat cells using the Amaxa nucleofection kit. The transfected cells were routinely checked for expression of p24 in the culture supernatant and for the appearance of syncytia. At the time large syncytia formed, the level of soluble p24 was 500 ng/ml. One ml of this culture medium was used to transmit the virus to a second culture of Jurkat cells to confirm the infectivity of the virus produced by the *in vitro* circularized 1-LTR$_{HIV}$, as shown. (C) Virus transmission from HeLa-tat cells transfected with *in vitro* circularized 1-LTR$_{HIV}$. Transfection was performed as described in (B). (D, E) Virus transmission from HeLa-tat cells transfected with (D) linear or (E) *in vitro* circularized 1-LTR$_{HIV}$ following indirect cocultivation, as described in (A) and (B).

Fig. S4. Cross-section of a transwell insert in a tissue culture plate used for indirect cocultivation of Jurkat cells with 1) human PBL transfected with 1-LTR$_{HIV}$, or 2) with HeLa-tat transfected with linear or circular forms of 1-LTR$_{HIV}$ as described in the legend of Fig. S3.

Fig. S5. Electron Microscopy analysis of HeLa-tat cells transfected with 1-LTR$_{HIV}$. Cells were prepared and analyzed using the procedure described in the Materials and Methods (procedure B) for adherent cells. Calibration=1.083 nm/pixel at 10.0k. Magnification: (A) x12.0k; (B) x 25.0k; (C) x10.0k; (D) x15.0k; (E) x10.0k. A 200nm scale bar is shown in each figure.

Table S1. Virus transmission to Jurkat cells. Transmission from (A) cocultivation of Jurkat cells with HeLa-tat cell transfected with 1-LTR$_{HIV}$ or from (B) Jurkat cells transfected with 1-LTR$_{HIV}$ using the experimental procedures reported in the legend to Fig. S2.

Fig. S1

A

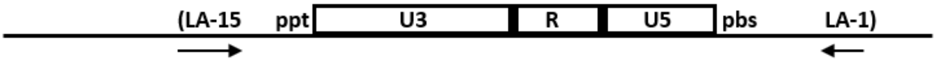


```
                (9005          LA-15
clone 1 fw    9005-CACACCTCAGGTACCTTTAAGACCAATGACTTACAAGGCAGCTGTAGATCTTAGCCACTT-9064
clone 1 rc    -------------------------------------------------AGATCTTAGCCACTT-9064

                         ppt           9084|1              5'-U3
clone 1 fw    9065-TTTAAAAGAAAAGGGGGGACTGGAAGGGCTAATTCACTCCCAACGAAGACAAGATATCCT-40
clone 1 rc    9065-TTTAAAAGAAAAGGGGGGACTGGAAGGGCTAATTCACTCCCAACGAAGACAAGATATCCT-40
                ************************************************************

                       5'-U5 |  NarI/pbs
clone 1 fw    627- CTCTAGCAGTGGCGCCCNAACAGGGACTT-----------------------------------655
clone 1 rc    627- CACTAGCAGTGGCGCCCGAACAGGGACTTGAAAGCGAAAGGGAAACCTGAGGAGCTCTCT-683

clone 1 rc     684- CGACGCAGGACTCGGCTTGCTGAAGCGC-711
                            LA-1            711)
```

B

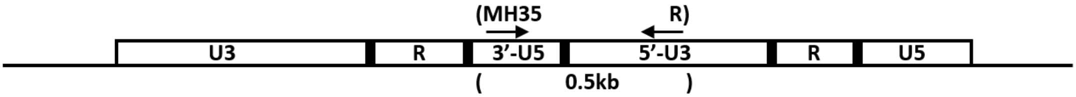


```
                      MH535
clone 1 fw   9585-AACTAGGGAACCCACTGCTTAAGCCTCAATAAAGCTTGCCTTGAGTGCTTCAAGTAGTGTGT
                  GCCCGTCTGTTGTGTGACTCTGGTAACTAGAGATCCCTCAGACCCTTTTAGTCAGTGTGGAA

              3'-U5    9718|Junction |1               5'-U3
             AATCTCTAGCAGTCCACTGGAAGGGCTAATTCACTCCCAACGAACAAGATATCCTTGATCTGT
             GGATCTACCACACACAAGGCTACTTCCCTGATTGGCAGAACTACACACCAGGACCAGGGATCA
             GATATCCACTGACCTTTGGATGGCGCTACAAGCTAGTACCAGTTGAGCCAGAGAAGTTAGAAG
             AAGCCAACAAAGGAGAGAACACCAGCTTGTTACACCCTGTGAGCCTGCATGGAATGGATGACC
             CGGAGAGAGAAGTGTTAGAGTGGAGGTTTGACAGCCGCCTAGCATTTCATCACGTGGCCCGAG
             AGCTGCATCCGGAGTACTTCAAGAACTGCTGATATCGAGCTTGCTACAAGGGACTTTCCGCTG-363
                                                    2-LTR rev.circle (R)
```

Fig. S2

A

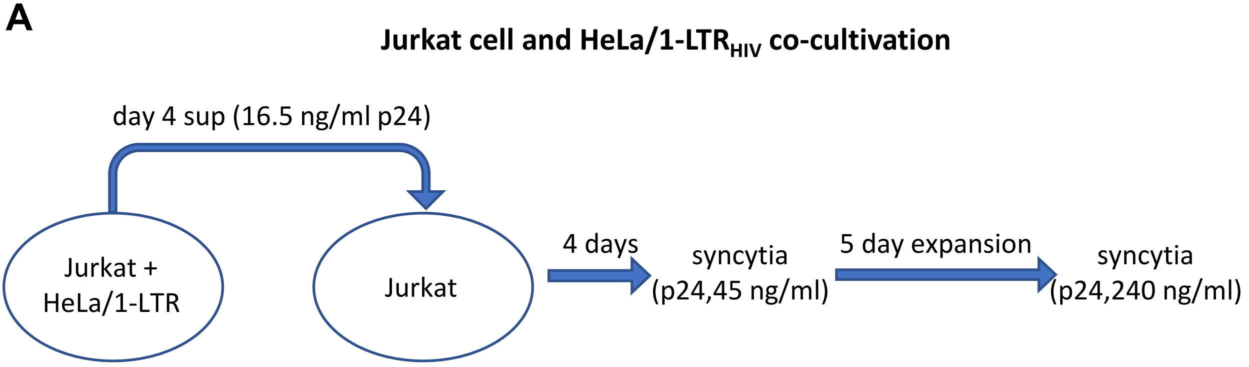
Jurkat cell and HeLa/1-LTR$_{HIV}$ co-cultivation
day 4 sup (16.5 ng/ml p24)
Jurkat + HeLa/1-LTR
Jurkat
4 days
syncytia (p24,45 ng/ml)
5 day expansion
syncytia (p24,240 ng/ml)

B

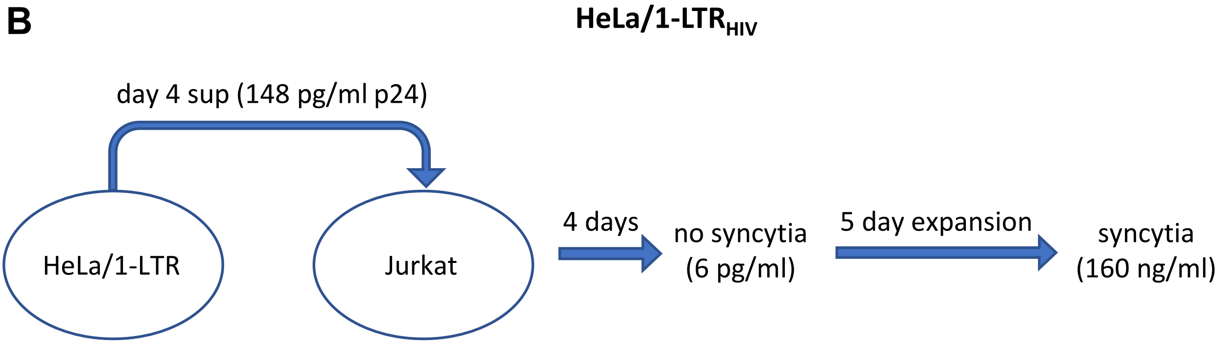
HeLa/1-LTR$_{HIV}$
day 4 sup (148 pg/ml p24)
HeLa/1-LTR
Jurkat
4 days
no syncytia (6 pg/ml)
5 day expansion
syncytia (160 ng/ml)

Fig. S3

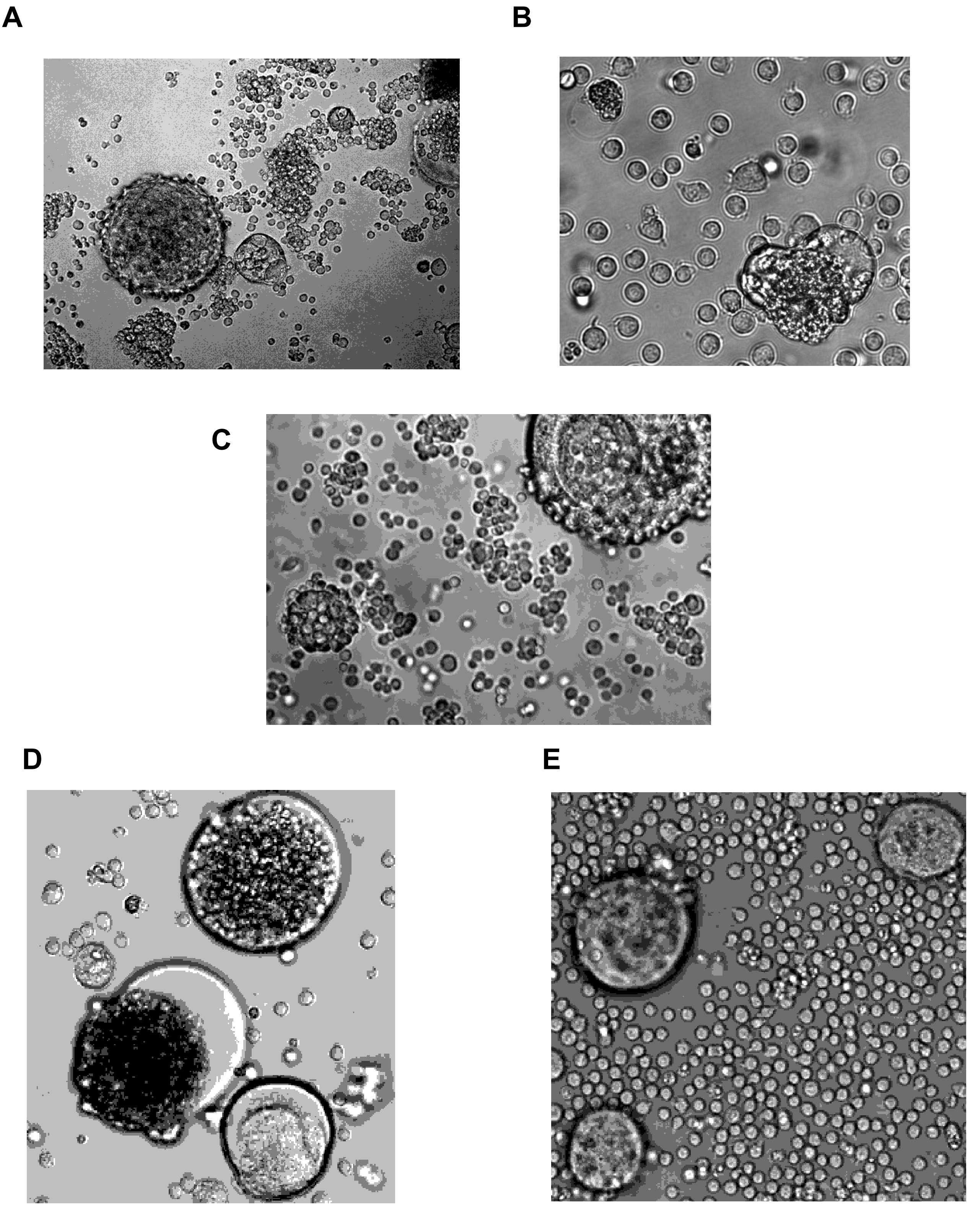

Fig. S4

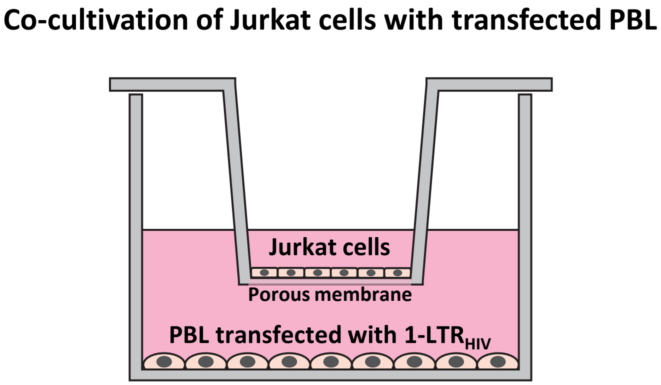
Co-cultivation of Jurkat cells with transfected PBL
Jurkat cells
Porous membrane
PBL transfected with 1-LTR$_{HIV}$

Fig. S5

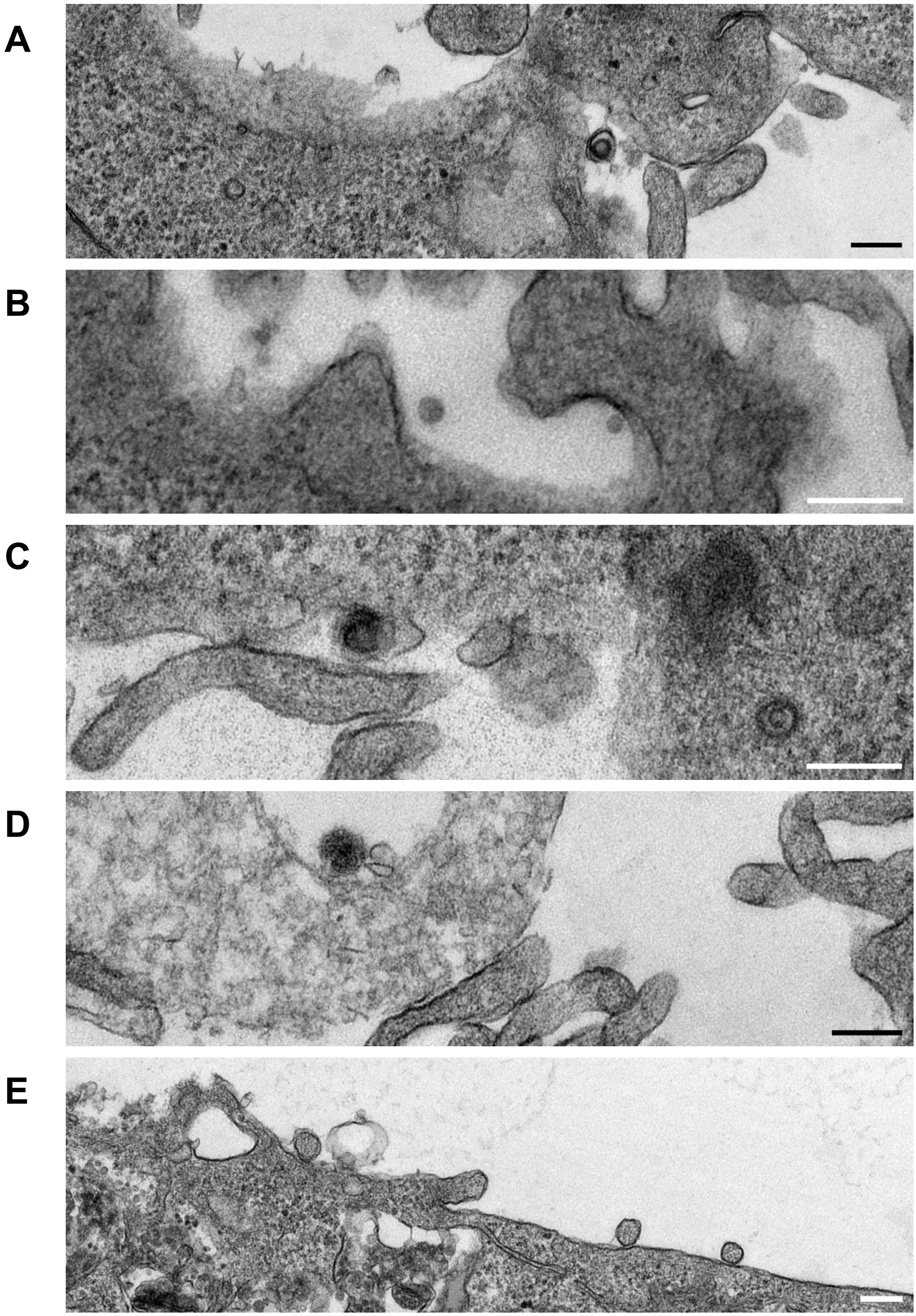

Table S1

**A**	**Cocultivation (4 days)**	**Transmission to:**	**4 d. after transmission**	**+5 d. expansion**
**cells**	Hela/1-LTR; Jurkat	Jurkat/2x10e6 cells		
**P24**	66 ng/4ml (cocult snt)	16 ng/1ml (transmitted)	45 ng/ml (cult snt)	240 ng/ml
**Syncytia**			Positive	Positive

**B**	**Cultivation (4 days)**	**Transmission to:**	**After transmission**	
**cells**	Hela/1-LTR	Jurkat/2x10e6 cells		
**P24**	0.4 ng/4ml (cult snt)	148 pg /1ml (transmitted)	6 ng/ml	160 ng/ml
**Syncytia**			Negative	Positive
