# Supplementary figures and images for: Expression of HIV from a 1-LTR circular DNA in the absence of integration
Source: Retrovirology. 2025 Mar 17;22:2. doi: 10.1186/s12977-025-00658-1 (PMC11912779; doi:10.1186/s12977-025-00658-1)

Fig. 3A

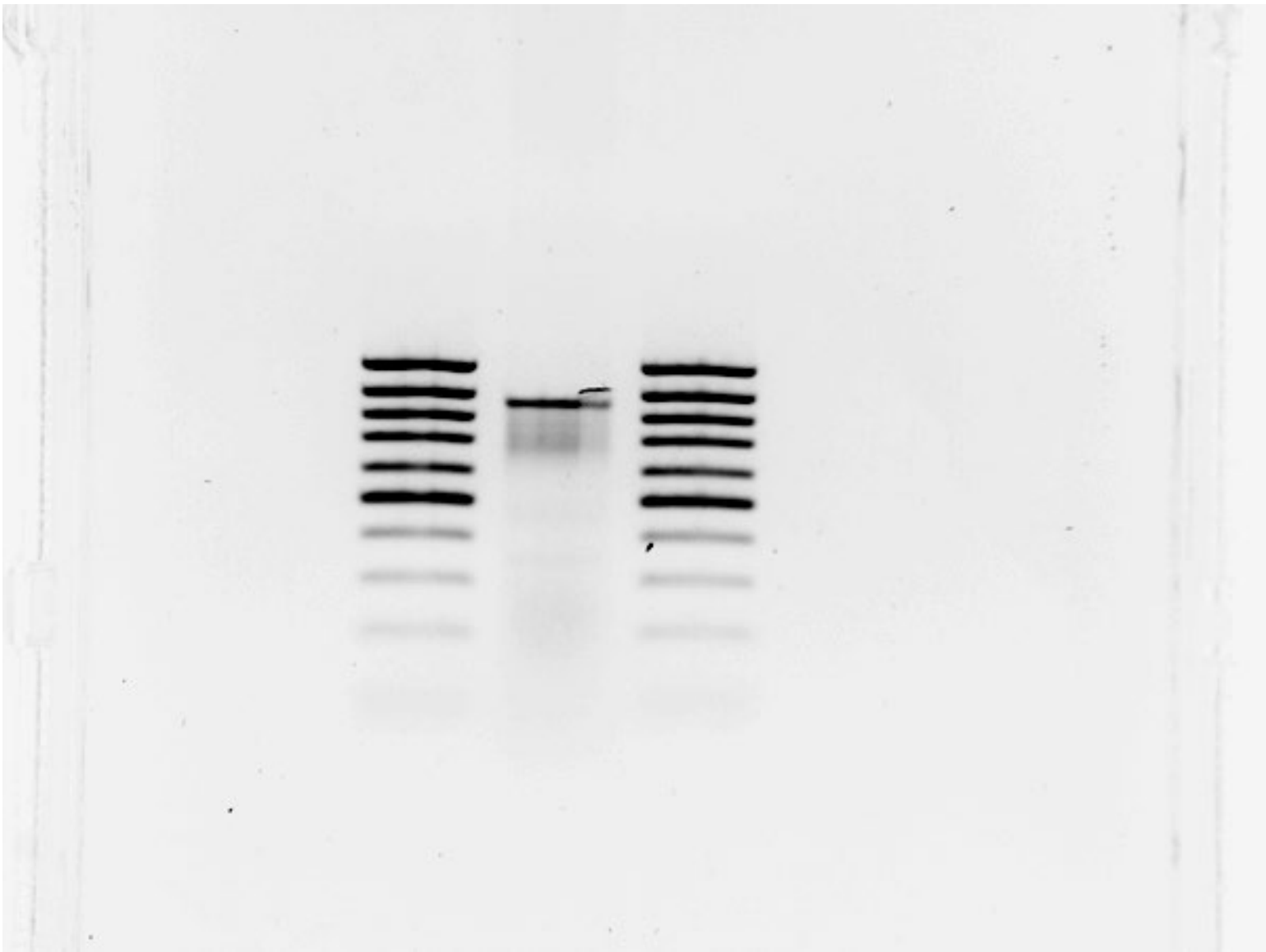

Fig. 4A

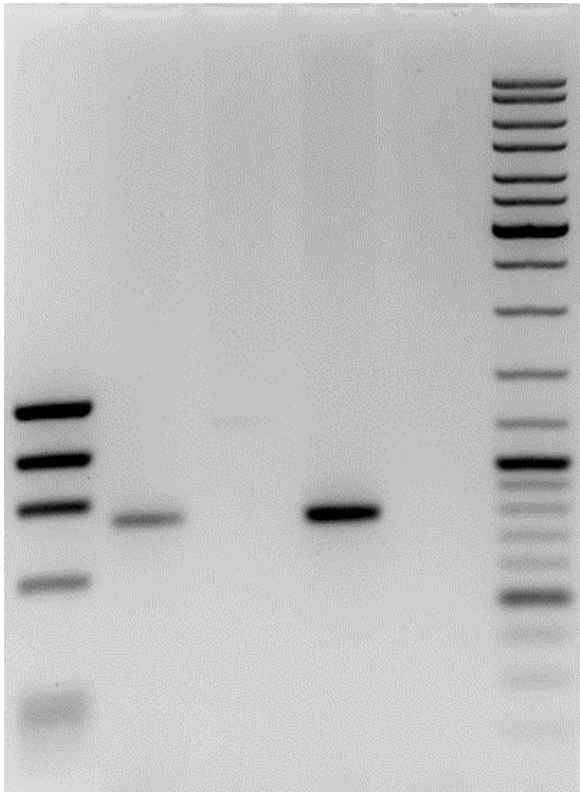

Fig. 4B

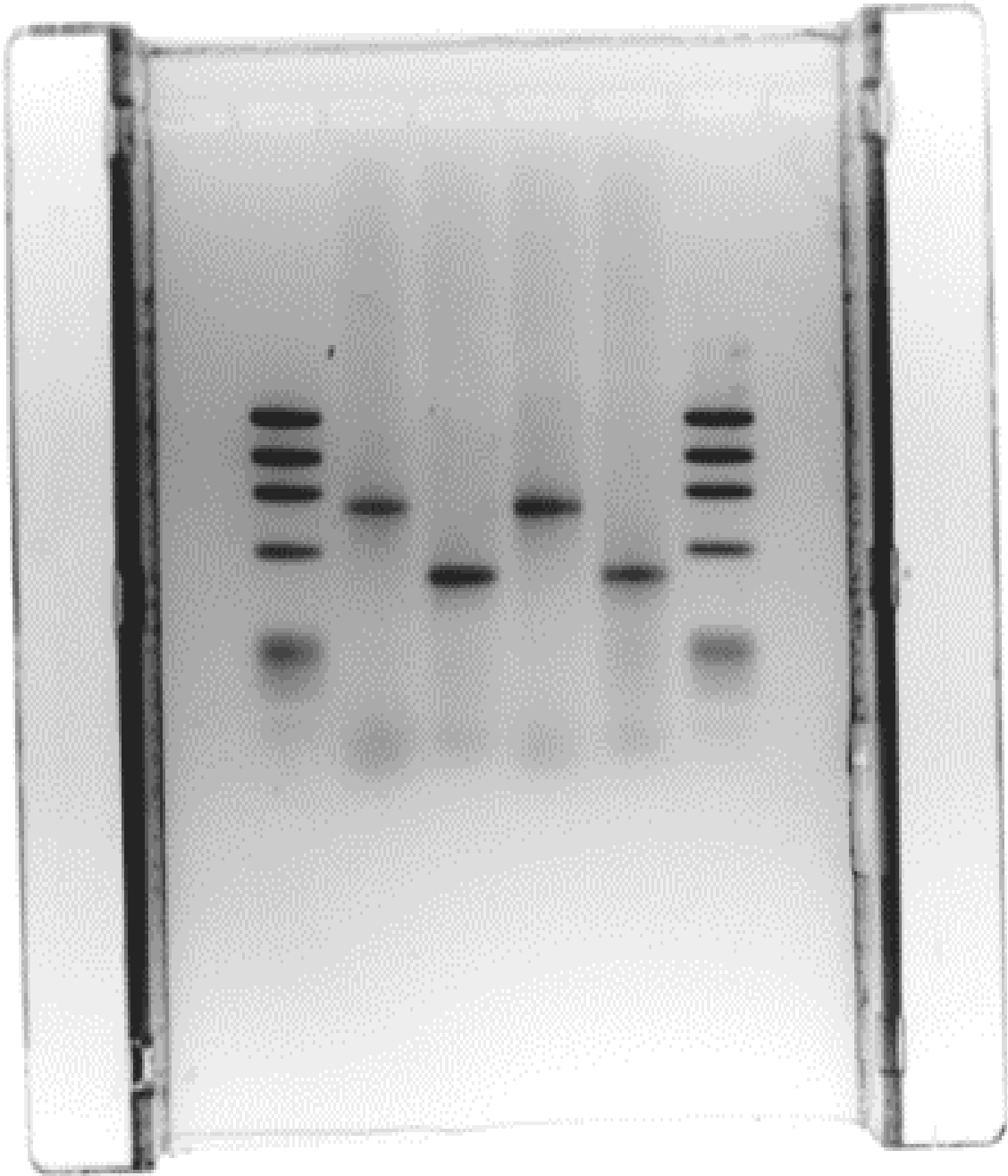

Supplement: Supplementary file 2 — Supplementary Material 2 [file 12977_2025_658_MOESM2_ESM.pdf]
